# Highly efficient magnetic ablation and the contrast of various imaging using biocompatible liquid–metal gallium

**DOI:** 10.1186/s12938-022-01003-9

**Published:** 2022-06-17

**Authors:** Chiang-Wen Lee, Ming-Hsien Chiang, Wen-Chun Wei, Shu-Shien Liao, Yen-Bin Liu, Kuan-Chih Huang, Kuen-Lin Chen, Wen-Cheng Kuo, Yuan-Ching Sung, Ting-Yuan Chen, Ju-Fang Liu, Yao-Chang Chiang, Hsin-Nung Shih, Kuo-Ti Peng, Jen-Jie Chieh

**Affiliations:** 1 Institute of Electro-Optical Engineering, National Taiwan Normal University, Taipei, Taiwan; 2 Department of Nursing, Division of Basic Medical Sciences, and Chronic Diseases and Health Promotion Research Center, Chang Gung University of Science and Technology, Chiayi County, Taiwan; 3 Department of Orthopedic Surgery, Chiayi Chang Gung Memorial Hospital, Chiayi County, Taiwan; 4 Department of Anatomy and Cell Biology, College of Medicine, National Taiwan University, Taipei, Taiwan; 5 Division of Cardiology, Department of Internal Medicine, National Taiwan University Hospital, Taipei, Taiwan; 6 Division of Cardiology, Heart Center, Cheng-Hsin General Hospital, Taipei, Taiwan; 7 Graduate Institute of Clinical Medicine, National Taiwan University, Taipei, Taiwan; 8 Department of Physics, National Chung Hsing University, Taichung, Taiwan; 9 Department of Mechanical and Automation Engineering, National Kaohsiung University of Science and Technology, Kaohsiung, Taiwan; 10 School of Oral Hygiene, College of Oral Medicine, Taipei Medical University, Taipei, Taiwan; 11 Department of Orthopaedic Surgery, Linkou Chang Gung Memorial Hospital, Taoyuan, Taiwan; 12 College of Medicine, Chang-Gung University, Taoyuan, Taiwan

**Keywords:** Magnetic ablation, Wireless, Self-degradation, Biosafety, Imaging

## Abstract

**Background:**

Although the powerful clinical effects of radiofrequency and microwave ablation have been established, such ablation is associated with several limitations, including a small ablation size, a long ablation time, the few treatment positioning, and biosafety risks. To overcome these limitations, biosafe and efficient magnetic ablation was achieved in this study by using biocompatible liquid gallium as an ablation medium and a contrast medium for imaging.

**Results:**

Magnetic fields with a frequency (*f*) lower than 200 kHz and an amplitude (*H*) × *f* value lower than 5.0 × 10^9^ Am^−1^ s^−1^ were generated using the proposed method. These fields could generate an ablation size of 3 cm in rat liver lobes under a temperature of approximately 300 °C and a time of 20 s. The results of this study indicate that biomedical gallium can be used as a contrast medium for the positioning of gallium injections and the evaluation of ablated tissue around a target site. Liquid gallium can be used as an ablation medium and imaging contrast medium because of its stable retention in normal tissue for at least 3 days. Besides, the high anticancer potential of gallium ions was inferred from the self-degradation of 100 µL of liquid gallium after around 21 days of immersion in acidic solutions.

**Conclusions:**

The rapid wireless ablation of large or multiple lesions was achieved through the simple multi-injection of liquid gallium. This approach can replace the currently favoured procedure involving the use of multiple ablation probes, which is associated with limited benefits and several side effects.

**Methods:**

Magnetic ablation was confirmed to be highly efficient by the consistent results obtained in the simulation and in vitro tests of gallium and iron oxide as well as the electromagnetic specifics and thermotherapy performance comparison detailed in this study Ultrasound imaging, X-ray imaging, and magnetic resonance imaging were found to be compatible with the proposed magnetic ablation method. Self-degradation analysis was conducted by mixing liquid gallium in acidic solutions with a pH of approximately 5–7 (to imitate a tumour-containing microenvironment). X-ray diffraction was used to identify the gallium oxides produced by degraded gallium ions.

**Supplementary Information:**

The online version contains supplementary material available at 10.1186/s12938-022-01003-9.

## Background

To promote survival and minimise side effects, localised therapies are being increasingly adopted for treating cancers [1]. Localised therapies are generally classified into two categories: those involving the injection of treatment materials (e.g., targeted drugs, chemicals, and hormones) [2–4] and those involving the use of electromagnetic energy (e.g., radio wave energy [5] and thermal energy [6–23]; Table 1). Low-temperature cryoablation involves the injection of treatment materials into the body [22, 23]. Local thermotherapy induces additional immunotherapy effects [2, 24]. Local thermotherapy can be of two types: (1) higher-temperature thermotherapy for conducting ablation (> 50 °C) [6–14] and (2) lower-temperature thermotherapy for inducing hyperthermia (41–46 °C) [16, 22] (Table 1). The protein coagulation time and recovery time can be rapid under ablation [25, 26]. For example, in previous studies, the protein coagulation time was 60 min at 46 °C or 4–6 min at 50–54 °C [6, 18]. In hospitals, postablation observation of specific organs requires only a few hours. After ablation, ablation-size-dependent transient flu-like symptoms and mild discomfort are always experienced and should be treated conservatively; however, unusual pain, swelling, and excessive bleeding should be treated immediately [27].

**Table 1:** Types of thermal-energy-based treatment

Dangerous energy	Thermal energy,,one of surgery (The temperature of treatment site vs. body temperature) [1]					Treatment materials	
Radiotherapy	Higher temp (so-called local thermotherapy)				Lower temperature	Chemical ablation, embolization	Chemo-, targeted, hormone, immuno-therapy
	Ablation (> 50 ℃ or 800 V/cm)		Hyperthermia (2–5 V/cm or 41 ~ 45℃)		Cryo-ablation (− 20 to − 40℃)		
[Noninvasive]	[Noninvasive] (LA, HIFU)	[Mini-invasive] (IRE,RF, MW)	[Noninvasive] (Electro-field)	[Mini-invasive] (MNPs)	[Mini-invasive]	[Mini-invasive]	
	(LA) < 100 μm size,800–1100 nm, 25 W; (HIFU) 1–5/20 MHz, 1,000 ~ 10,000 W/cm^2^	(IRE)3000 V,50A, 250/500 kHz; (RF)350–500 kHz (MW)900–2500 MHz	13.56 MHz, 40 W-150 W, 60/80 min. for several times	0.02 − 1.1 MHz, 2–20 kAm^−1^, tens of minutes	− 140 ℃/ − 190 ℃, double freeze–thaw cycles (13–35 min.)		
• Many side effects • Combination with thermo-therapies [5]	• Surface or shallow depth • Limited treatment size/time (LA: 2 cm for 10 min; HIFU: 3.5 cm) [6, 7]	• Several hours wait for IRE impact • Limited treatment size (< 3 cm) /time [8–14]	• Limited tumor specificity • Combination with chemo/ radiotherapies [15–17]	• Limited to the regions without spreading away • Applied fields with healthy-risk specifics against 200 kHz or 5.0 × 10^9^ am^−1^ s^−1^ [18–21]	• The inability to control hemorrhage without intra-arterial access [22, 23]	• The inability to control position • Limited treatment effect • Combination with thermo-therapies [3, 4]	• Systematic treatment • Large individual difference • Induced/enhanced by thermo-therapies [2, 3]

**Fig. 1:**
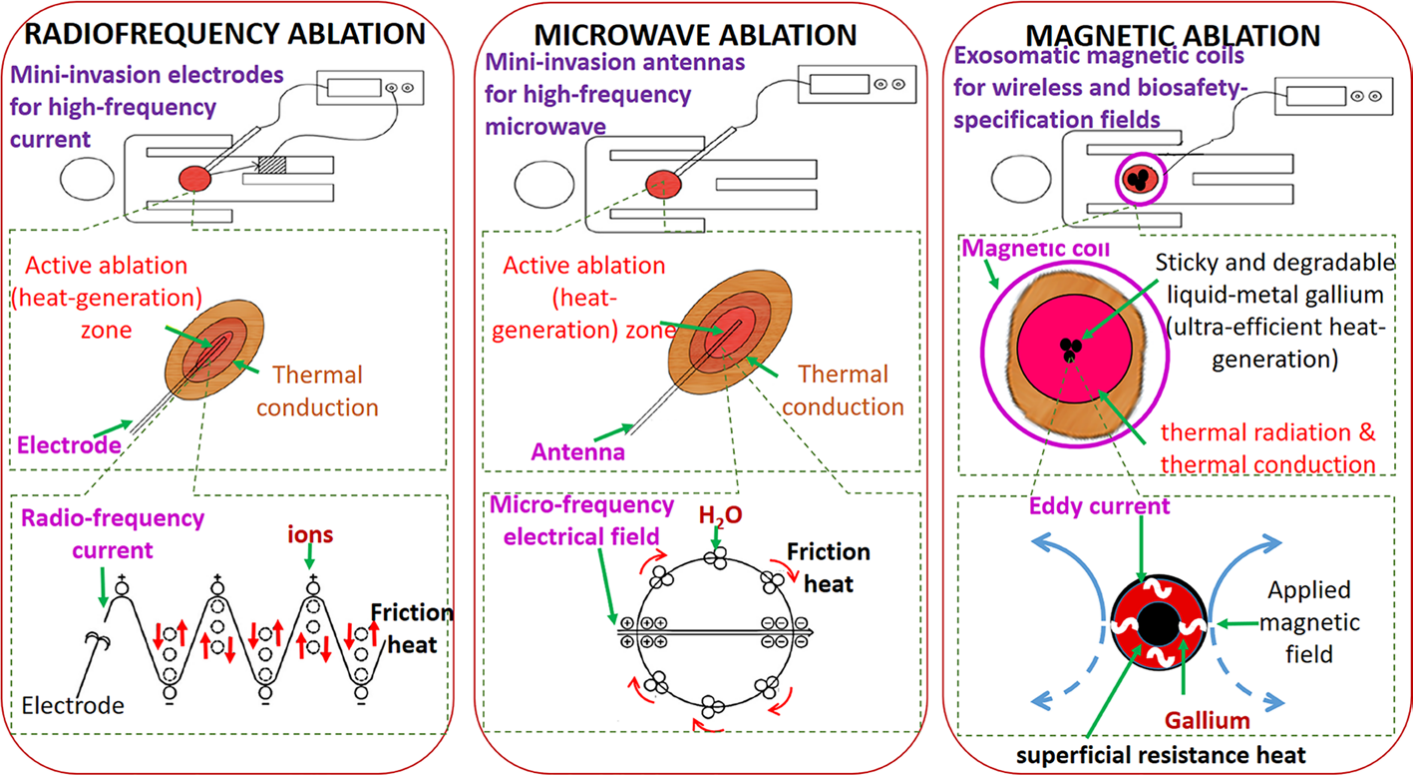
Schemes and mechanisms of RF ablation, MW ablation, and the proposed magnetic ablation method. The upper part of this figure displays the schematic of the adopted system. The middle part illustrates the process of tissue classification by temperature. In RF and MW ablation, the high-temperature active ablation zone and low-temperature thermal conduction zones are located around the electrode and antenna, respectively. By contrast, in the proposed magnetic ablation method, only the high- and low-temperature thermal conduction zones are located near the high-temperature active ablation zone of the injected gallium. The bottom part of this figure illustrates the mechanism in the high-temperature active ablation zone. In RF and MW ablation, friction heat is generated through ion movement and water molecule rotation. In magnetic ablation, surface resistance heat is generated from the high-speed flow of eddy current in the superficial region of the conductive materials

In thermotherapy, the temperature depends on two major factors: the heat generation efficiency and heat dissipation caused by blood circulation. The heat generation efficiency refers to the efficiency with which designated regions absorb and convert emitted power into therapeutic heat, such as acoustic waves as high-intensity focused ultrasound (HIFU) [7], electromagnetic waves as laser ablation [6] and microwave (MW) ablation [12–14], radiofrequency (RF) currents [10, 11], electric fields as irreversible electroporation [8, 9] and electro-field hyperthermia [15–17], and magnetic fields as magnetic nanoparticle (MNP)-induced hyperthermia [15, 19–21] (Table 1). An inefficient heat generation mechanism cannot overcome biological heat dissipation. Therefore, even using the risk electromagnetic specifics over the biosafety criteria, the volume of heated tissue is still limited, and heating time over the thermal homeostasis criterion is risk. For an exposure region with a diameter smaller than 30 cm, the frequency of the generated magnetic field (*f*) should be less than 200.0 kHz, and the product of the intensity of the field (*H*) and *f* should be less than 5.0 × 10^9^ Am^−1^ s^−1^ [21, 28, 29]. Another biosafety criterion is the thermal homeostasis criterion, which is approximately 5–7 min in humans to avoid side effects [30, 31]. The side effect of the thermal homeostasis phenomenon is diversion of heat away from the thermotherapeutic region under physiological feedback through blood flow once the neighbouring tissue has been heated [32]. Thermal homeostasis not only limits the rate of temperature increase and the maximum temperature reached but also induces cancer cell metastasis, because the generated heat leaves the ablated region through blood vessels [30]. One study indicated that cancer cell metastasis, rather than in situ carcinoma, shortens the survival of patients with cancer to within a few years after treatment completion [11]. RF ablation and MW ablation involve the inefficient heating of tissue within a diameter of 2–3 cm over 10–12 or 8–10 min using high-frequency waves or current0 (Table 1; Fig. 1) [10–14]. Therefore, several large or discrete lesions have been reported to occur when multiple RF or MW ablation probes with high power consumption have been employed [13, 14]. Moreover, in HIFU ablation, scanning with a single, small, and high-power focus induces the biosafety risks of an excessively long thermotherapy duration [7, 33] and fibrosis [7]. Visualisation of a treatment is often crucial, and such visualisation is achieved through methods including magnetic resonance imaging (MRI)-guided HIFU [33] and ultrasound-guided percutaneous injection [10].

The trend has shifted from the development of heat generation mechanisms on the basis of tissue materials or characteristics to the development of biocompatible materials for local and efficient thermotherapy and auxiliary imaging [34]. Large or discrete tumours can be imaged by injecting several biocompatible imaging contrast media into them [35]. Large or discrete lesions can be heated using a wireless and exosomatic electromagnetic generator (the rightmost example in Fig. 1). This phenomenon is highly likely to mitigate the biosafety risks posed by the use of multiple RF or MW ablation probes or scanning with a single high-power HIFU focus. For example, iron oxide MNPs are commonly used for performing MRI imaging and achieving MNP-induced hyperthermia, which involves the use of alternating current (AC) magnetic fields [15–21]. However, the limited heat generation efficiency of MNP-induced hyperthermia results in tissue being heated only at the hyperthermia temperature level between 40 to 50 degrees for a long duration (approximately tens of minutes), worsen than the ablation temperature level over 50 degrees for the short ablated time over the minutes, within the thermal homeostasis criterion (approximately 5–7 min). Therefore, several researchers have increased the heat generation efficiency using nonbiocompatible materials with favourable magnetic properties or applying AC magnetic fields associated with the aforementioned biosafety risks [19–21, 28, 29]. Moreover, MRI imaging with MNPs can be used for positioning but cannot be used for analysis of ablated tissue because of the distortion of uniform direct current (DC) fields by MNPs. In one study, the low signal-to-noise ratio in MRI imaging was adequate for positioning; however, the tissue’s condition could not be accurately determined [33].

This paper proposes the use of liquid gallium, which has excellent biocompatible and conductive properties, to achieve biosafe, wireless, and highly efficient magnetic ablation (the rightmost example in Fig. 1). Liquid gallium can be used as a contrast medium in several popular imaging modalities. A minute quantity of pure gallium is found in the human body, where it has unclear physiological functions. Moreover, gallium has well-known influences on cellular processes and biologically crucial proteins, especially those produced through iron metabolism [36–38]. For example, gallium’s influence on iron homeostasis leads to phenomena, such as disruption of ribonucleotide reductase, mitochondrial function impairment, and regulation of transferrin receptor and ferritin. Consequently, gallium affects iron-dependent proliferation processes in tumour cells by functioning as an iron mimetic. The binding of gallium to siderophores and the downregulation of bacterial iron uptake cause the disruption of iron utilisation by microbes, which results in iron having anti-infective activity against bacteria and fungi [39]. Therefore, gallium ions become bound and concentrated in areas of inflammation and rapid cell division [40] in various biomedical applications [41]. For example, gallium citrate is used as a contrast medium for the radioactive imaging of some cancers [42, 43]. Moreover, it has potential utility as artificial neural networks [41] as well as therapeutic effects against certain cancers [36] and infectious microorganisms [39]. Gallium-based liquid metals exhibit higher biocompatibility and biodegradability than do conventional metal compounds, whose ions, such as Gd^3+^ and Mn^2+^, can be acutely toxic to organs [44]. Some studies have discovered the gallium metabolism pathways in numerous organ systems, such as the liver, kidneys, skeleton, bone marrow, and spleen. Gallium can reach these organ systems through transferrin [45] and routes of excretion from urine, faeces, and breast milk [38, 39, 45]. The safe dose of an intravenous gallium nitrate infusion is approximately 900 mg/m^2^ for a brief infusion [39]. In contrast to the self-degradation of gallium in the weakly alkaline environments of normal tissue [46], the self-degradation of gallium in weakly acidic cancer environments remains unclear. Thus, the degradation of gallium in different acidic solutions and its retention in nonacidic tissue were examined in this study.

## Results

### Heat generation mechanism of magnetic ablation

**Fig. 2:**
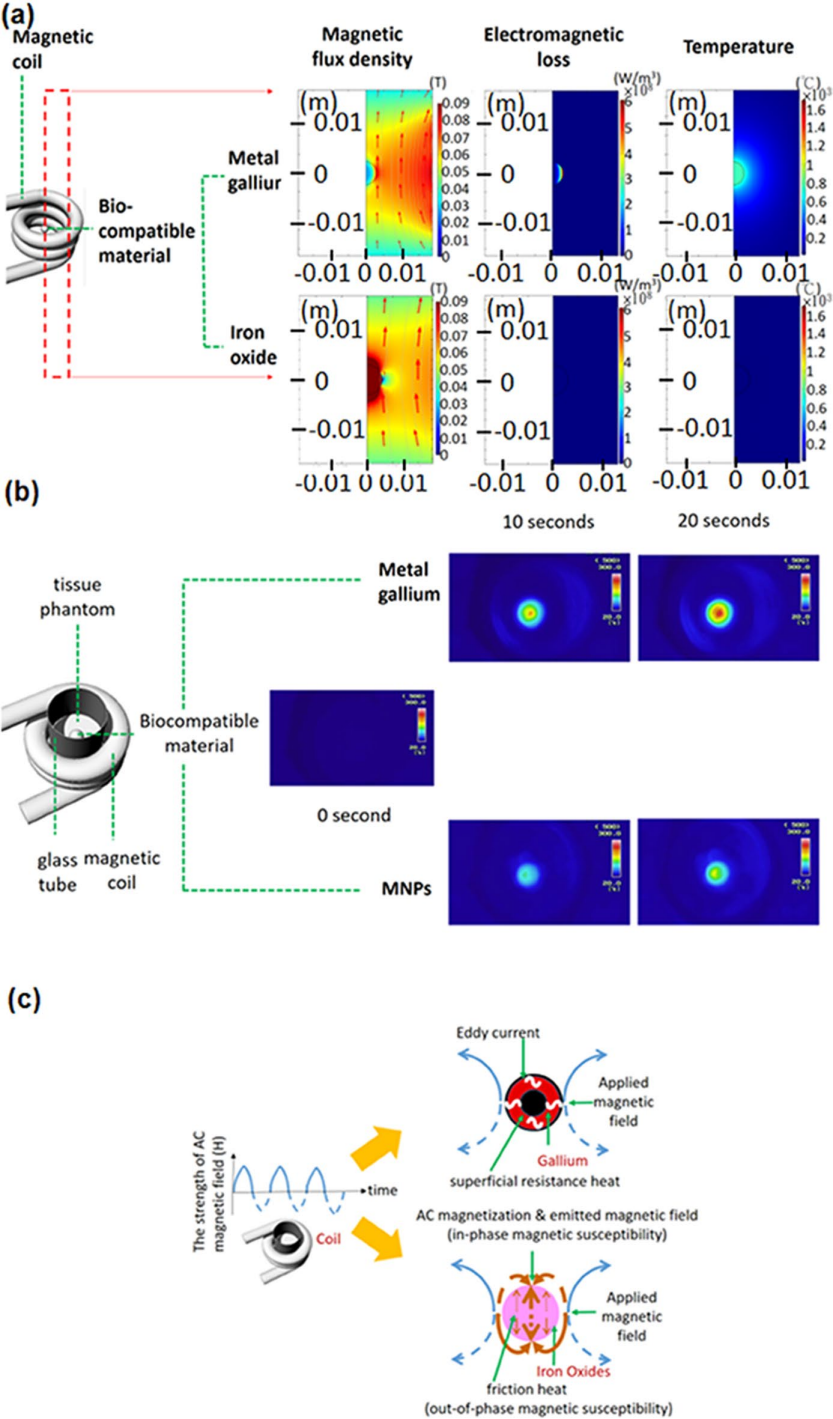
Simulation and experimental results for the heat generated by 65-μL samples of gallium and magnetic iron oxides. **a** The magnetic ablation mechanism was examined under different values of magnetic flux density, electromagnetic loss, and ablation temperature at a fixed ultrashort ablation time of 10 s. **b** Experimental temperature values were obtained for ablation times of 0, 10, and 20 s. **c** Schematic of the heat generation mechanism in magnetic thermotherapy when injecting gallium and magnetic iron oxides into tissue

Studies on the efficient heat generation mechanism in magnetic ablation have presented simulation results on three crucial parameters of magnetic flux density, electromagnetic loss, and ablation temperature for various types of biocompatible material (e.g., gallium and magnetic iron oxides) at the 10th second, since the application of magnetic fields (Fig. 2a). Liquid gallium possesses high conductivity for several biomedical applications [41].

The primary results obtained in this study were the magnetic flux density around magnetic coils and test materials under an AC magnetic field (*f* = 37.2 kHz, *H* = 46,652.63 Am^−1^; Fig. 2a and Additional file 1: Fig. S3a). In the space surrounding the gallium, the magnetic flux gradually decreased towards the centre from the coil boundary. However, the magnetic flux was abnormally stronger (higher than 0.09 T) at the outer spot around the rightmost material (represented by the tiny red arc in Fig. 2a) than at the uppermost and lowermost spots. The flux decreased from the coil towards the magnetic iron oxides except at ultrahigh and ultralow flux levels at the top or bottom spaces and in the rightmost material. The magnetic fluxes in the inner half-circle differed considerably among different materials. For gallium, the inner magnetic flux rapidly decreased in an arc distribution from 0.08 T at the right boundary to 0.02 T at the semicircular centre. For the iron oxides, a uniformly ultrahigh flux of 0.09 T was observed.

The secondary results obtained were the electromagnetic loss within the heat-generating materials of gallium and iron oxides (Fig. 2a). Heat was defined as any power loss, rather than converted stored energy. The electromagnetic loss of gallium decreased from 6 × 10^8^ to 0 × 10^8^ W/m^3^ in a thin and colourful arc in the semicircular rightmost area in Fig. 2a. However, magnetic iron oxide particles with loss smaller than 1 × 10^8^ W/m^3^ were difficult to observe. The tertiary results were the temperature within the ablated material. The temperature within the semicircular area of the material and in the neighbouring space uniformly exceeded 700 °C for gallium; however, the temperature for the magnetic iron oxides was approximately 120 °C. Infrared images of the in vitro testing revealed that the average temperature within the maximum-temperature region increased from 213 °C at the 10th second to almost 300 °C at the 20th second (Fig. 2b: upper panel) for all quantities of gallium (Additional file 1: Fig. S3b). For the magnetic iron oxides, the temperature increased from 117 °C at the 10th second to nearly 158 °C at the 20th second (Fig. 2b: lower panel).

### Ablation size and time in rat livers with rich blood circulation

**Fig. 3:**
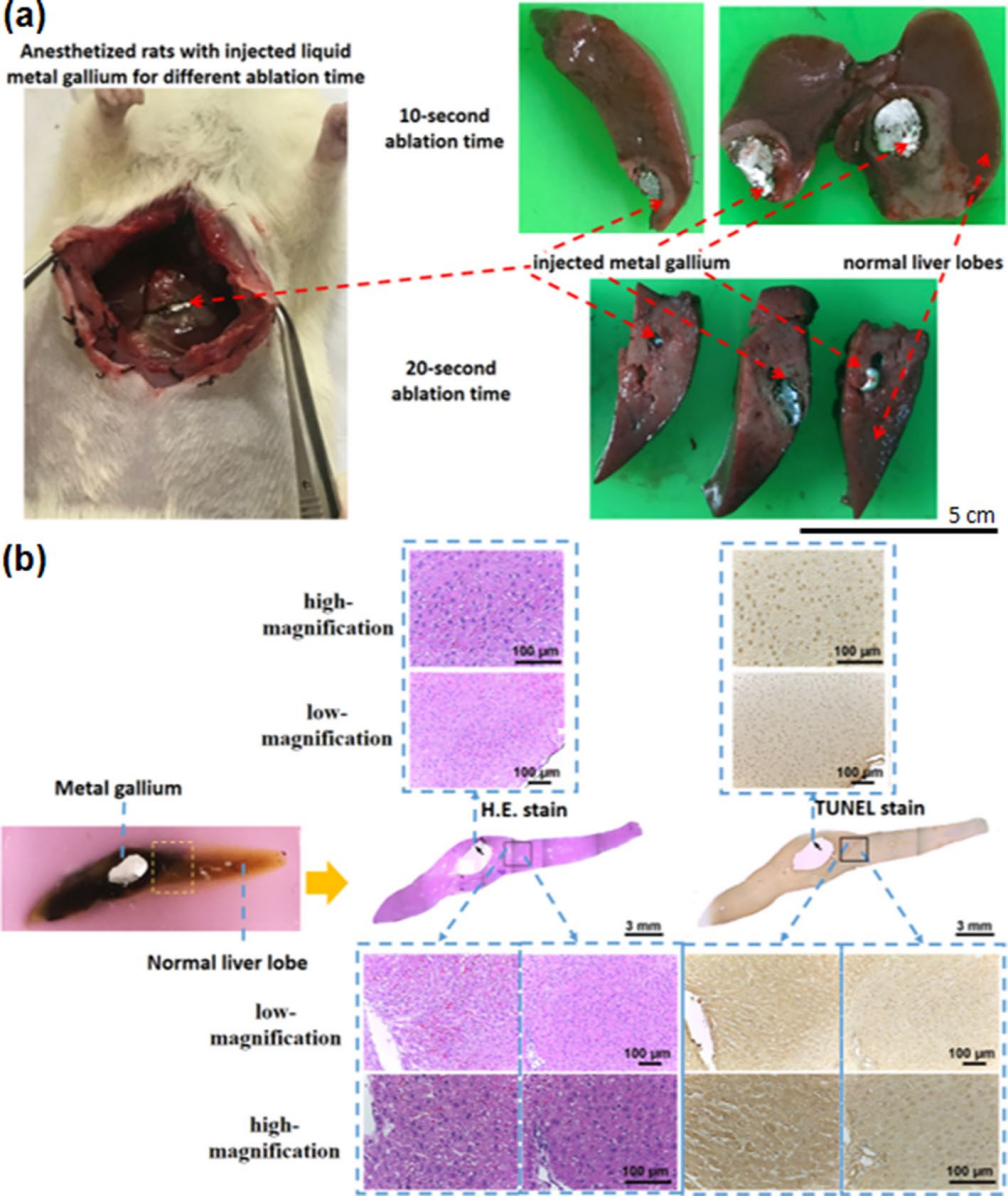
Ablation sizes and times. **a** Healthy liver lobes of anaesthetised rats with rich blood circulation were injected with gallium through an incision (left). The ablation size was larger 20 s after injection than 10 s after injection (right). **b** Photographs indicate healthy hepatic tissue after 10 s of magnetic ablation (left: wax block before H&E and TUNEL staining, right: stained tissue). The right images display the entire stained tissue (middle part) and local tissue (upper and lower parts) at the surface of the gallium (arrow) and the boundaries between ablated and normal regions (in boxes). The regions observed under high magnification are indicated by dotted blue rectangles

For biological heat transfer loads greater than those in in vitro testing, the same volume of liquid gallium was injected through an abdominal incision into several healthy and high-circulation liver lobes of anaesthetised rats (Additional file 1: Fig. S4). The absence of any magnetic fields surrounding the injected gallium in the 5 min was used as a reference standard. Subsequently, the rat abdomens were placed within a magnetic coil under an AC magnetic field (*H* = 46,652.63 Am^−1^, *f* = 37.2 kHz) for various ablation times. On the day after ablation, no abnormal behaviours were noted in the anaesthetised rats. The injected gallium exhibited a single-spot distribution in the livers, and no special distribution pattern or leakage was observed (Fig. 3a: left panel).

The ablation size caused by the gallium spots on the six lobes was 5 ± 2 mm after an ablation time of 10 s, and the ablation size had increased to 3.8 ± 0.8 cm (i.e., marginally larger than half a lobe) after an ablation time of 20 s. For an embedding wax of a liver lobe block, the boundary separating the live and ablated tissues was near the remaining gallium after an ablation time of 10 s (Fig. 3b: left panel). Haematoxylin and eosin (H&E) staining and terminal deoxynucleotidyl transferase deoxyuridine triphosphate (dUTP) nick-end labelling (TUNEL) staining were performed on the liver lobes (Fig. 3b: right panel). Microscopic images of the central region of the liver lobes (Fig. 3b: right panel) revealed that this region had the same profile as that displayed in a photograph of the wax block (Fig. 3b: left). This profile did not contain the hole of liquid metal during staining. The distance of the ablated boundary from the hole was approximately ≥ 2 mm after an ablation time of 10 s, which indicated that the ablation resolution was finer than 1 mm. The ablation resolution could be adjusted by manipulating the ablation time or field strength. The black arrow (Fig. 3b: right) points towards the ablated tissue situated adjacent to the location from which liquid metal was removed. High- and low-magnification microscopy images (Fig. 3b: upper and lower panels, respectively) revealed the existence of red blood cells in the liver lobes. These cells are indicated by a dark red colour in the aforementioned images. Therefore, regarding the two parts of the black box (Fig. 3b) across the boundary, the left part presented the same red blood cells with respect to the positive results as those of the ablated tissue, whereas the right part presented negative results.

### Compatibility with various imaging methods

**Fig. 4:**
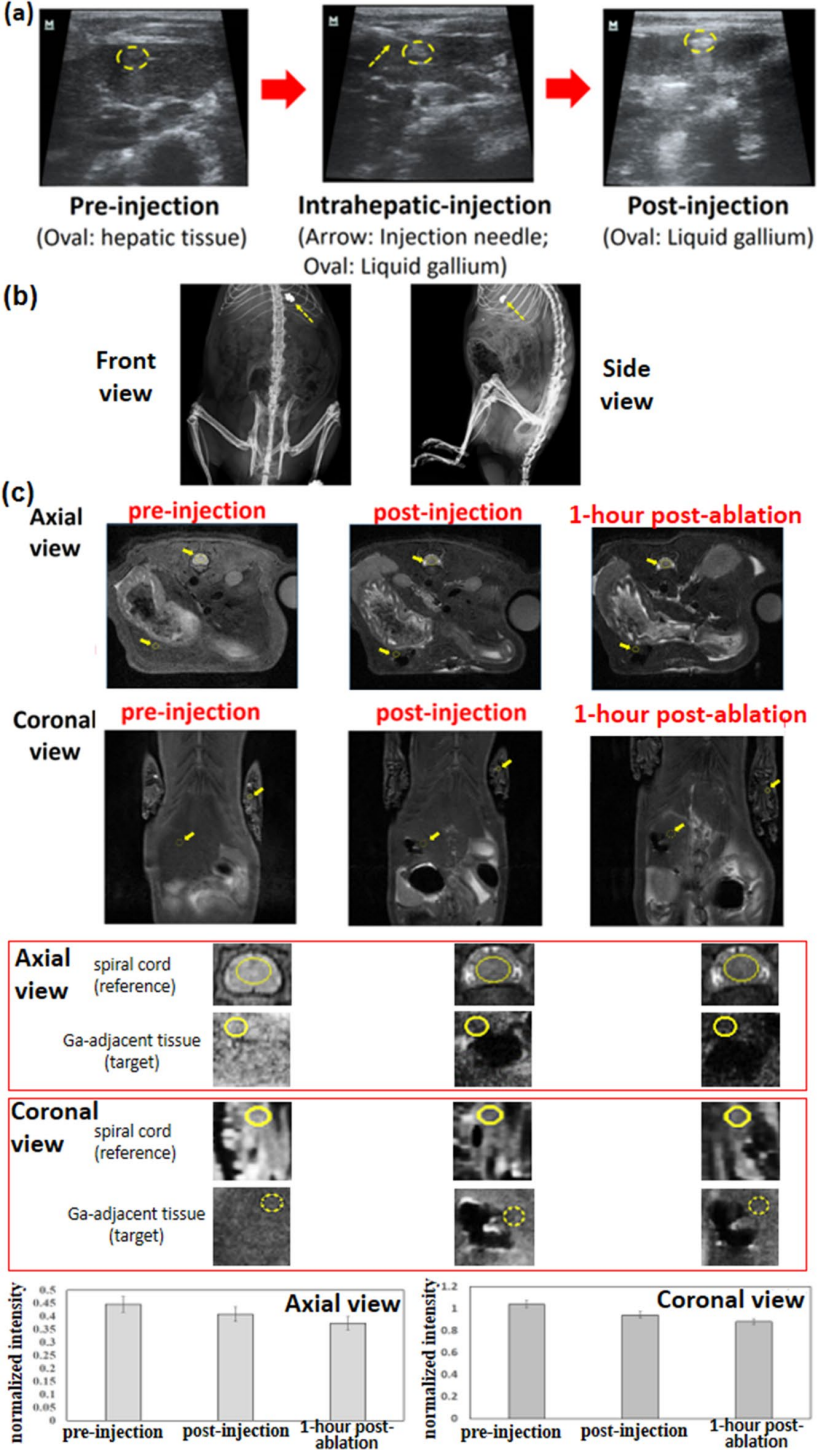
Images obtained through different methods. **a** The positions of a mini-invasive needle and the injected gallium are determined through ultrasonic imaging. **b** The position of the injected gallium was determined through X-ray imaging (front and side views). **c.** The position of the injected gallium was determined, and the tissue was analysed before injection, after injection, and after ablation through MRI tomography (axial and coronal views). The ablation time was 10 s

Ultrasound images of the liver lobes, captured before the gallium injection, exhibit no white spots; those captured during the hepatic injection of gallium exhibit white spots corresponding to the invasion needle (yellow arrow) and injected gallium (yellow oval); and those captured after gallium injection exhibit white spots corresponding to gallium (Fig. 4a). Regarding the treatment positioning (Fig. 4b), the injected gallium could be observed clearly through X-ray imaging (i.e., a type of density-difference-based ultrasound imaging). In some thermotherapy regimens, MRI tomography based on hydrogen atoms is suitable for evaluating, positioning, and guiding the treatment. Tissue without containing water can also be imaged through MRI tomography (Fig. 4c). The tissue near the injection region in the left part of the abdomen of the rats is marked by yellow circles with a diameter of approximately 1 cm in in the axial and coronal views of the abdomen (Fig. 4c). Prior to gallium injection, the aforementioned tissue was light grey. After gallium injection and 1 h after ablation, a large black spot was observed on this tissue because of the injection of black gallium. Ablation was conducted under an AC magnetic field. A comparison of the states of the tissue near the injection region before the injection, after the injection, and 1 h after the ablation indicated that the tissue became progressively darker with injection and ablation.

To validate the aforementioned phenomenon without the interference of measurement drift, the normalised intensity was considered the image intensity ratio of the yellow circles in the target tissue over those in reference locations (e.g., the MRI intensity of the spinal cord or foot bones as the mentioned reference location are negligibly affected by the circulated materials and body temperature). In one of the rats, the normalised intensity (which had a small standard error in the coronal or axial view) decreased because of gallium injection or tissue ablation (Fig. 4c).

### Challenges involved in the retention of injected materials by and the ablation of the main vessel tissue of active muscles on the surface of a rat’s leg

**Fig. 5:**
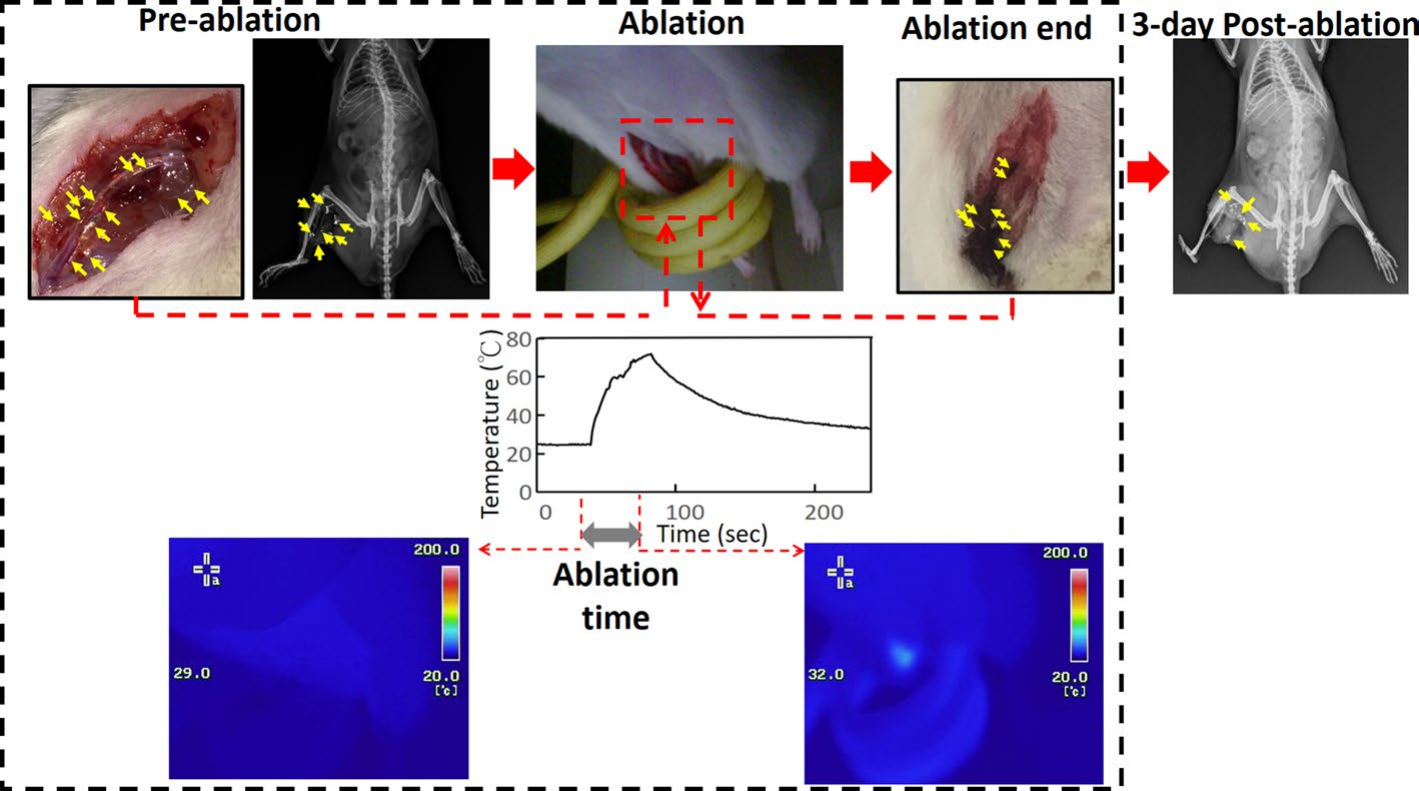
Tissue retention of gallium. Before ablation, liquid gallium was injected into the active leg muscle along the main vessels of the rats, as shown in the photographs and X-ray images (yellow arrow). After the legs of the anaesthetised rats had been placed within a magnetic coil, the temperature of the tissue injected with gallium and the studied domain were investigated using a fibre-optic thermometer and infrared imaging during the ablation. The tissue colours and the positions of gallium retention (determined through 3-day X-ray imaging, yellow arrow) before and after ablation were compared

The stable retention of injected materials by tissue is the foremost requirement for ablation and imaging. Two animal tests indicated that magnetic ablation (Fig. 3) and various types of imaging (Fig. 4) could be conducted and the several-hour retention of injected materials could be achieved in a high-circulation hepatic environment. However, organ vibration and a low heat transfer load under anaesthetic conditions limited the retention of injected materials through rat abdomens. A total of 100 μL of gallium was injected into the active muscles on the leg surface of the rats (the muscles most frequently used for running) between the high-circulation main vessels (the added environment cooling into biological heat transfer as the more serious challenge). In the ablation involving the insertion of rat legs within the magnetic coil, the tissue temperature had increased to 70 °C after 39 ± 2 s under the AC magnetic field (as indicated by the attached fibre-optic thermometer). When the magnetic field was turned off, the temperature gradually returned to 26 ± 0.3 °C, which was comparable to the initial temperature. Approximately 34 ± 4 mm of the ablated tissue surface became dark, as indicated by the postablation photographs. A comparison of the photographs and X-ray images of the ablated tissue at different times (Fig. 5) indicated that the locations of the ablation end and gallium content 3 days after ablation were nearly the same as those before ablation.

### Self-degradation

**Fig. 6:**
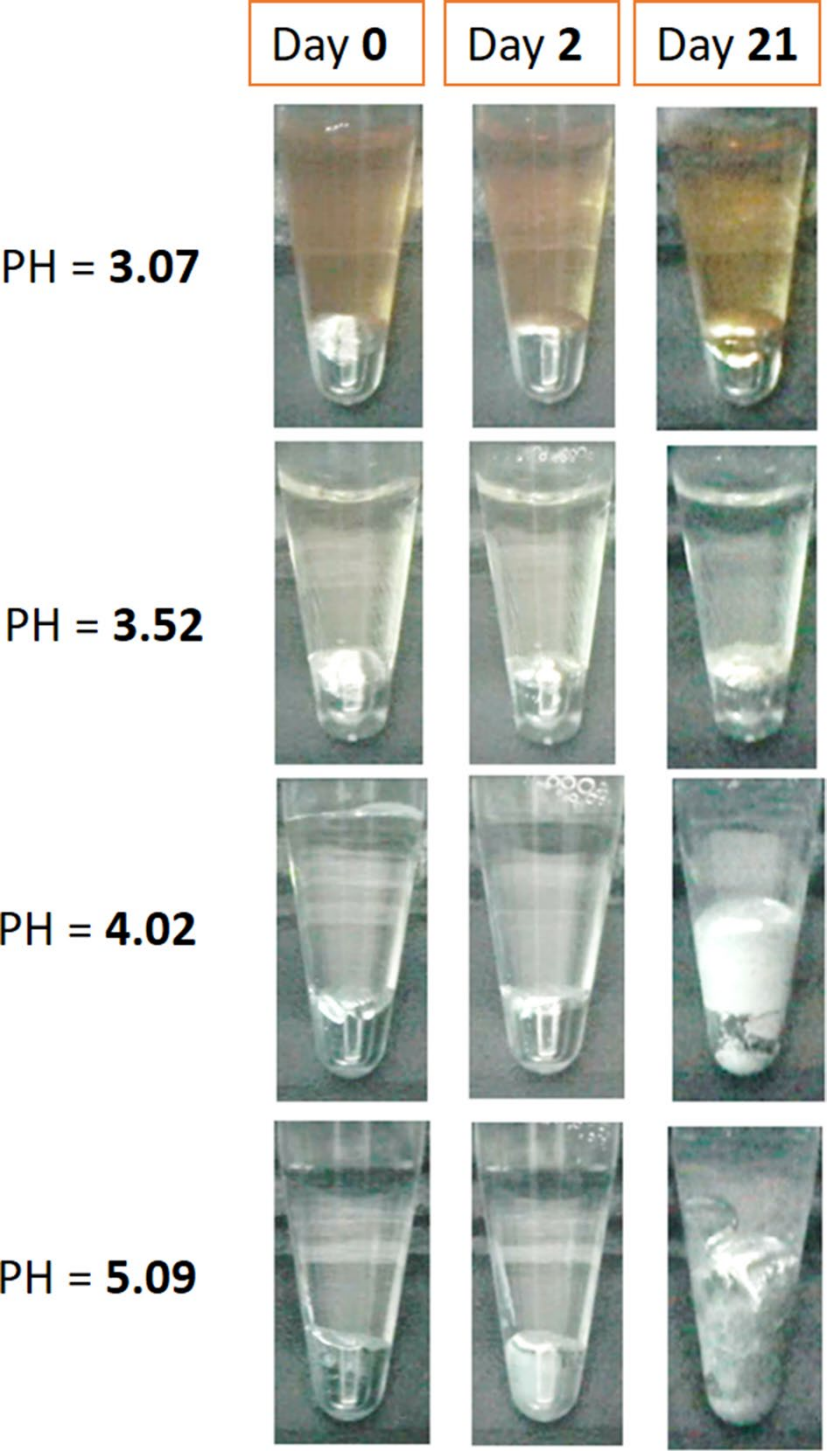
Degradability of gallium. The degradability of the injected gallium in magnetic ablation was determined by adding it to solutions with pH values of 3.07, 3.52, 4.02, and 5.09 immediately, 2 days, and 32 days after ablation

Self-degradation away from the target tissue is an essential requirement for injected materials. At pH values of 3.07, 3.52, and 4.02, gallium did not degrade after 2 days; however, it exhibited different degradation levels after 21 days (Fig. 6). For example, a dark surface appeared above the shiny metal at a pH of 3.07. A thin white layer appeared above the dark grey metal at a pH of 3.52, and a thick white layer appeared above the space containing white spots and the dark grey metal at a pH of 4.02. By contrast, at the highest pH of 5.09, the gallium had become grey after 2 days had passed, and a thin white layer had formed above the space containing white spots and a dark grey layer by day 21. The white-spot powder was identified as gallium oxide hydroxide (GaOOH), which is the immediate precursor of Ga_2_O_3_ (Additional file 1: Fig. S5).

## Discussion

Using the results of a simulation and an in vitro experiment (Fig. 2a, b, Additional file 1: Fig. S3), the heat generation efficiency was evaluated to determine the heat generation mechanisms of gallium and magnetic iron oxides (Fig. 2c). The mechanisms of the conductive gallium and magnetic iron oxides are as follows. The distribution of the electromagnetic loss of gallium (an intensive, superficial, and arc-shaped distribution; Fig. 2a) was in agreement with that of the eddy current flow of gallium under superficial resistance heat (Fig. 2c). Here, positive and negative half-cycle AC magnetic fields generated by the coil are denoted by solid and dashed lines, respectively (Fig. 2c), and shielded by the alleged skin depth. Hence, the magnetic field flux (marked in black) in the deeply inner of a gallium particle was lower that the surface (Fig. 2c). The spatial distribution of magnetic fields agreed with the variation in the magnetic flux density (Fig. 2a). The magnetic absorption power of the iron oxides contributed greatly to the magnetisation of magnetic materials because of the large in-phase AC susceptibility. In Fig. 2c, thick arrows in the same direction as the applied magnetic field denote the direction of the in-phase AC susceptibility. Some of the magnetic absorption power was converted into heat; however, because of the low out-of-phase AC susceptibility. In Fig. 2c, the direction of the out-of-phase AC susceptibility is indicated by thin arrows in the opposite direction to the applied magnetic field. The low out-of-phase AC susceptibility was consistent with the low electromagnetic loss of the magnetic iron oxides (Fig. 2a, in which the electromagnetic loss distribution of the magnetic iron oxides is displayed at the same scale as that of gallium).

These results were fairly representative of both thermotherapy and imaging using the same energy source. For example, the large in-phase AC susceptibility of MNPs in the MRI of high-DC magnetic fields induces the strong self-magnetisation of these particles, and the uniform distortion of local DC fields around an MRI coil results in MNP-containing tissue exhibiting black spots in T_2_-weighted MRI images [47]. Therefore, evaluating the ablation level on the basis of the MRI intensity is difficult in MNP-based thermotherapy. In this study, tissue near the injection site exhibited a smaller reduction in the normalised MRI intensity after the injection of gallium than after the injection of MNPs (Fig. 4c), which indicated that the injected gallium caused a smaller distortion in the local DC magnetic field [48] than did the injected MNPs; thus, the injected gallium functioned as a marker of the ablation of neighbouring tissue. The small reduction in the normalised intensity after ablation when injecting gallium indicated that the ablated tissue was located near the injection site (revealed by the protein denaturation or moisture loss in T2-weighted MRI images). For example, a small distance existed between the target tissue (the yellow circle) and the black spot at which gallium was injected, as indicated by the ablation size being 5 ± 2 mm for an ablation time of 10 s in the tissue staining results (Fig. 3). Overall, the magnetic ablation performance and MRI contrast in this study were favourable. The high density of gallium means it can be used as a contrast medium in density-based imaging (ultrasound and X-ray imaging) for guiding or positioning the treatment locations (Fig. 4a, b).

The conversion of a minor part of the magnetic absorption power into heat by MNPs indicated the low heating temperature achieved in MNP-induced hyperthermia. Moreover, the rate of temperature increase for a heat generation material depended on the effective heat (the difference between the generated and dissipated heat) and specific heat capacity of the material. For example, the dried MNPs in the in vitro testing (Fig. 2b) had considerably higher temperature than did the MNPs in the aqueous solutions [18–21] because of the high specific heat capacity of water. Ultrahigh temperatures were observed for gallium (Fig. 2b), and these temperatures are attributed to the fact that gallium has considerably lower specific heat capacity (367 J g^−1^ K^−1^) than does water [49]. Ultrahigh temperatures often occur in metal cutting, which is a common industrial application. Furthermore, in RF and MW ablation, in which electromagnetic energy (RF current or MWs) is converted into heat, the high specific heat capacity of water-containing tissue (3620 J g^−1^ K^−1^) limits the increase in temperature of the tissue [50].

Similar to the small magnetic energy generated by MNPs under AC magnetic fields, the friction heat produced through ion movement or water molecule rotation accounted for an ultrasmall portion of the absorbed electromagnetic power in RF or MW ablation (Fig. 1). The kinetic power of the water-based materials accounted for most of the kinetic power generated and increased with the frequency. The friction heat generally increased with the kinetic power and thus the frequency. Therefore, frequencies exceeding the biosafety frequency criterion of 200 kHz (Table 1) were used in RF and MW ablation even when *f* × *H* exceeded 5.0 × 10^9^ Am^−1^ s^−1^, and higher ablation performance was achieved in high-frequency MW ablation than in RF ablation. For the ablation size of 3 cm in the in vivo livers, the ablation time and ablation temperature were, respectively, 8–10 min and 90 °C in MW ablation and 10–12 min and 70 °C in RF ablation (Fig. 1) [10–14]. The aforementioned ablation times exceeded the biosafety criterion of 5–7 min for the ablation time. However, the magnetic ablation under the magnetic field met the biosafety criteria for *f* and *f* × *H* (*f* = 37.2 kHz and *f* × *H* = 1.73 × 10^9^ Am^−1^ s^−1^), and the gallium in air achieved high temperature of 300 °C at 20 s after the injection (Fig. 2; Additional file 1: Fig. S3b). Thermal radiation and thermal conduction are assumed to be the major and minor heat transfer mechanisms of the ultra-high-temperature gallium, respectively, because these heating phenomena are completed within several seconds (Fig. 1). The heating time was 20 s for in vivo liver lobes with an ablation size of 3.8 ± 0.8 cm and no tissue carbonisation (Fig. 3), and the heating time was 39 ± 2 s for shallow leg muscles with an ablation size of 34 ± 4 mm and apparent tissue carbonisation (Fig. 4). Although these short heating times meant there was no risk of cancer metastasis [14], the environment acted as a heat sink and the high circulation slowed the tissue carbonisation and the temperature increase, which was 70 °C after 39 ± 2 s.

Finally, because of its high viscosity (2.13 × 10^−3^ Pa S at 30 °C) [51], the injected liquid gallium was stable in the tissue and did not break down due to the interstitial gap or torso activity. Therefore, the injected gallium remained in the injection region for at least 3 days (Fig. 5). Consequently, the injected gallium was used as a marker and an image contrast medium for evaluating the ablation level (Fig. 4). However, a quantity of gallium that was at least equal to the injection volume in the animal tests degraded into GaOOH through the reaction of Ga^3+^ ions with water molecules (supplement Fig. 5) over time and with changes in the pH of the acidic solution. In the most representative case, the shiny gallium immersed in the solution with a pH of 5.09 became white on day 2 and grey on day 21. By contrast, the colour barely changed in the strongly acidic solutions (Fig. 5). According to the corrosion mechanism of gallium, the enhancement and suppression of gallium degradation are dependent on the concentrations of water and salt, respectively [52]. The acidity of a tumour microenvironment increases with the number of tumours [46, 53]; thus, gallium may degrade sooner when an environment contains a higher number of tumours. Gallium is degraded in a weakly acidic environment in the presence of few tumours; however, in contrast to the degraded iron ions created through MNP-induced hyperthermia, which are nutritional irons for tumour growth [54], degraded gallium ions (i.e., the predecessors of gallium oxides) have anticancer properties [36]. Thus, drug treatments are possible following magnetic ablation. Consequently, magnetic ablation with liquid metals, in which wireless ablation probes self-degrade, is superior to current electromagnetic ablation approaches, in which ablation probes must be manually removed.

## Conclusions

In this study, magnetic ablation was performed under the biosafety specifications of AC magnetic fields by injecting liquid gallium into tissue under high temperatures within an ultrashort time and with highly efficient heat generation. The feasibility of various imaging methods—ultrasound imaging, X-ray imaging, and MRI imaging or positioning—for evaluating tissue ablation levels was examined. The stable retention of liquid gallium in normal tissue facilitated these biomedical functions of imaging and ablations. The dependency of the self-degradation of gallium on the pH of a solution indicated the high potential of gallium (in metallic and ionic forms) for antitumour treatments, including ablation and drug treatments.

## Methods

### Simulation testing

The COMSOL Multiphysics AC/DC module (COMSOL, Burlington, VT, USA) was used to determine the mechanism of magnetic ablation without considering the complexities of heat transfer in the experimental conditions. The generated magnetic field had a spherical shape with a diameter of 5 mm (volume of approximately 65.4 μL), and this field was located at the centre of a 40-mm-diameter magnetic coil with three turns (Additional file 1: Fig. S1: left part). The copper pipe of the magnetic coil had an inner diameter and outer diameter of 4 and 10 mm, respectively. Consequently, the three-turn magnetic coil was 40 mm in inner diameter, 60 mm in outer diameter, and 32 mm in height, respectively. The simulated air box had a height and diameter of 100 mm. Because the study domain exhibited spatial symmetry, only half the study domain is presented in Fig. 2a (left panel, dashed red line), and mesh-based calculations were performed for only half the study domain (Additional file 1: Fig. S1: right part).

The adopted material settings and algorithm models are described in the following text. The relative permeability and permittivity were 1 for air, the copper coil, and gallium. The real part of the complex relative permeability, imaginary part of the complex relative permittivity, and relative permittivity were 10, 1.7, and 1, respectively, for iron oxides. The electric conductivity was 6.76 × 10^6^, 100, and 0 Sm^−1^ for gallium, iron oxides, and air, respectively [48, 49]. For other parameters—such as the density, thermal conductivity, and constant-pressure heat capacity of the aforementioned materials—the preset values in utilized software were retained. The software algorithm was divided into two parts. The first part involved the use of Ampere’s law with 1000-A current in coils and a ball, and the other part involved the modelling of heat transfer in a solid at a temperature of 293.15 K and with a heat transfer coefficient of 5 Wm^−2^ K^−1^. The highest *f* × *H* value occurred when using an AC magnetic field generator under a capacitance of 69.7 nF (Additional file 1: Fig. S2). The strength *H* and frequency *f* of the AC magnetic field were set as 46,652.63 Am^−1^ and 37.2 kHz, respectively, and their product (*f* × *H*) was 1.73 × 10^9^ Am^−1^ s^−1^, smaller than the safety criterion of 5 × 10^9^ Am^−1^ s^−1^. The overall ablation time was 10 s, and the time step was 1 s.

### In vitro testing

The AC magnetic field generator was a modified high-frequency induction heating machine (SP25A, Shenzhen Shuangping Power Supply Technologies Co. Ltd., Shenzhen, China). It contained self-developed capacitor modules (Additional file 1: Fig. S2) with capacitance values of 55.7 and 69.7 nF. The first type of in vitro test involved placing 65-μL samples of gallium (5 N, Kunshan Zhangpu Town Weiju Trading Firm, Kunshan, China) and Fe_3_O_4_ (CAS. No 1317619, Sigma-Aldrich, USA) individually into a 5-mm-diameter circle on tissue phantoms inserted in a 5-cm-diameter coil centre under an AC magnetic field (*H* = 46,652.63 Am^−1^ and *f* = 37.2 kHz; Fig. 2b). The tissue phantoms comprised approximately 33.5% w/w boric acid, 35.8% w/w guar gum, 22.9% w/w water, 6.7% w/w polyacrylamide, and 1.1%w/w triglyceride.

The second type of in vitro testing involved placing different volumes (59, 118, or 177 μL) of liquid gallium in a glass tube (5.9 mm in outer diameter; Sinosun Impex, China) and examining the gallium’s temperature response under different strengths (*H*) and frequencies (*f*; *H*_1_ = 13,810.87 A/m at 49 kHz, *H*_2_ = 14,001.26 A/m at 47.7 kHz, and *H*_3_ = 15,231.84 A/m at 46.5 kHz) of the AC magnetic field generated using the capacitor modules (Additional file 1: Fig. S3a, b).

### Animal testing

All the experimental procedures used for animal testing followed the recommendations of the Guide for the Care and Use of Laboratory Animals (National Institutes of Health Publication No. 86–23, revised in 1985) and were approved by the Animal Care and Use Committee of Chang Gung University of Science and Technology. Male Sprague–Dawley rats (BioLASCO, Taiwan) that were aged 12 weeks were anaesthetised using 1–2.5% isoflurane (Isoflurane USP, Piramal Critical Care, Bethlehem, PA, USA; Additional file 1: Fig. S4) [47, 55]. In the animal tests, the AC magnetic field had a strength of 46,652.63 Am^−1^ and a frequency of 37.2 kHz.

First, the ablation range was modulated by varying the ablation time to determine the suitable ablation time range under the general size limitation of the target organs. Supine rats were positioned on a plastic boat, and for each rat, the abdomen was inserted within the magnetic coil under the same AC magnetic field for all rats (*H* = 46,652.63 Am^−1^ and *f* = 37.2 kHz) for magnetic ablation. Six anaesthetised rats were subjected to regular physical examination after they were placed for 60 s within the coil without any injection. Subsequently, gallium (65 μL) was injected into the two liver lobes of each rat through abdominal incision using a 27-gauge needle. After 5 min of preablation observation, the aforementioned rats were divided into two groups of three for respective 10 and 20 s of ablation. These rats were then sacrificed 1 day after the ablation, and their liver tissue was stained. A long postablation time was adopted for some observations, such as when observing rat behaviour or the stable distribution of injected gallium from a possibly fluid state under high-temperature heat generation.

Second, the compatibility of the following three imaging methods with the adopted magnetic ablation method was examined: MRI scanning (7 T MRI scanner, Biospec 70/30, Bruker, Ettlingen, Germany), X-ray imaging (P-B-125, Hitachi Inc., Tokyo, Japan), and ultrasound imaging (DP-50 Vet, Shenzhen Mindray Bio-Medical Electronics, China). Moreover, to investigate the finely ablated tissue, MRI tomography was conducted before injection, after injection (i.e., preablation), and 1-h after ablation. The ablation time in the MRI tomography was 10 s, and the MRI intensity was analysed using ImageJ software (v. 1.52a, National Institutes of Health, Bethesda, MD, USA). Furthermore, the injection was guided by performing ultrasound imaging before, during, and after the injection. In this method, liquid gallium (65 μL) was percutaneously injected into one liver lobe each of three rats. Only one liver lobe of each rat was studied, because studies have not yet verified whether gallium interferes with imaging.

Third, for the validation of stable retention characteristics, liquid gallium (100 μL) was injected into one of two rats along the boundary between the femoral artery and the femoral vein. X-ray imaging was performed 3 days after the magnetic ablation to monitor the in vivo distribution of liquid gallium. The temperature during magnetic ablation was measured through infrared imaging (Thermo GEAR G120EX, Nippon Avionics Co., Ltd., Tokyo, Japan), and a nonmetallic fibre-optic thermometer (OTG-280, OPSens Inc., Québec City, Canada) was connected to the relevant tissue to determine the temperature of the injected gallium.

Once all the animal testing procedures were complete, the rats were sacrificed 1 day after ablation.

### Tissue staining

H&E staining was performed to determine the histological morphology of the investigated tissue, and TUNEL staining was conducted to detect apoptosis. Paraffin-embedded tissue sections were sectioned and quantified using an in situ apoptosis detection kit (In Situ Cell Death Detection Kit, Roche, CA, USA). These sections were incubated with a TUNEL reagent in a dark chamber at 37 °C, and six nonoverlapping regions of each tissue section were imaged.

### Degradation testing

Lactic acid solution (dl-lactic acid solution, 80% purity, chemPUR Feinchemikalien und Forschungsbedarf GmbH, Karlsruhe, Germany) was diluted to different multiples of the original concentration in deionised water in accordance with the production of tumour cells [42, 43]. Subsequently, 100-µL samples of liquid gallium were added to 500-µL solutions with a pH of 3.07, 3.52, 4.02, or 5.09 in 1.5-mL microcentrifuge tubes (Eppendorf AG, Hamburg, Germany). The tubes were then observed using cameras for 21 days. The identification of gallium powder degradation was validated through X-ray diffraction analysis (JCPDF 00-054-0910, Additional file 1: Fig. S5).

## Supplementary Information

**Additional file 1.** NIH

## Data Availability

The data sets analysed during the current study are available from the corresponding author on reasonable request.

## Abbreviations

HIFU: High-intensity focused ultrasound
LA: Laser ablation
MW: Microwave
RF: Radiofrequency
IRE: Irreversible electroporation
MRI: Magnetic resonance imaging
MNP: Magnetic nanoparticles
AC: Alternating current
DC: Direct current
HE: Haematoxylin and eosin
TUNEL: Terminal deoxynucleotidyl transferase dUTP nick end labelling
