# Correction: Highly efficient magnetic ablation and the contrast of various imaging using biocompatible liquid–metal gallium

**DOI:** 10.1186/s12938-022-01017-3

**Published:** 2022-07-20

**Authors:** Chiang-Wen Lee, Ming-Hsien Chiang, Wen-Chun Wei, Shu-Hsien Liao, Yen-Bin Liu, Kuan-Chih Huang, Kuen-Lin Chen, Wen-Cheng Kuo, Yuan-Ching Sung, Ting-Yuan Chen, Ju-Fang Liu, Yao-Chang Chiang, Hsin-Nung Shih, Kuo-Ti Peng, Jen-Jie Chieh

**Affiliations:** 1 Institute of Electro-Optical Engineering, National Taiwan Normal University, Taipei, Taiwan; 2 Department of Nursing, Division of Basic Medical Sciences, and Chronic Diseases and Health Promotion Research Center, Chang Gung University of Science and Technology, Chiayi County, Taiwan; 3 Department of Orthopedic Surgery, Chiayi Chang Gung Memorial Hospital, Chiayi County, Taiwan; 4 Department of Anatomy and Cell Biology, College of Medicine, National Taiwan University, Taipei, Taiwan; 5 Division of Cardiology, Department of Internal Medicine, National Taiwan University Hospital, Taipei, Taiwan; 6 Division of Cardiology, Heart Center, Cheng-Hsin General Hospital, Taipei, Taiwan; 7 Graduate Institute of Clinical Medicine, National Taiwan University, Taipei, Taiwan; 8 Department of Physics, National Chung Hsing University, Taichung, Taiwan; 9 Department of Mechanical and Automation Engineering, National Kaohsiung University of Science and Technology, Kaohsiung, Taiwan; 10 School of Oral Hygiene, College of Oral Medicine, Taipei Medical University, Taipei, Taiwan; 11 Department of Orthopaedic Surgery, Linkou Chang Gung Memorial Hospital, Taoyuan, Taiwan; 12 College of Medicine, Chang-Gung University, Taoyuan, Taiwan

## Correction: BioMedical Engineering OnLine **21**, 38 (2022) https://doi.org/10.1186/s12938-022-01003-9

Following publication of the original article [1], the author noticed an error in the affiliation of Hsin-Nung Shih and Kuo-Ti Peng in the author group and a typo in the author name Shu-Hsien Liao.

Author name ‘Shu-Hsien Liao’ was incorrectly written as ‘Shu-Shien Liao’.

The affiliation ‘College of Medicine, Chang-Gung University, Taoyuan, Taiwan’ for Author Hsin‑Nung Shih is missing.

The affiliation detail for Author Kuo‑Ti Peng was incorrectly given as 'College of Medicine, Chang-Gung University, Taoyuan, Taiwan' but should have been 'Department of Orthopedic Surgery, Chiayi Chang Gung Memorial Hospital, Chiayi County, Taiwan’.

The original article [1] has been updated.
