# Supplementary material for: Highly efficient magnetic ablation and the contrast of various imaging using biocompatible liquid–metal gallium
Source: Biomed Eng Online. 2022 Jun 17;21:38. doi: 10.1186/s12938-022-01003-9 (PMC9205100; doi:10.1186/s12938-022-01003-9)
Supplement: Supplementary file 1 — Additional file 1. NIH [file 12938_2022_1003_MOESM1_ESM.docx]

**Supplementary material**

- **Simulation testing**

The COMSOL Multiphysics AC/DC module (COMSOL, Burlington, USA) was used to determine the mechanism of magnetic ablation without the inference of the complexities of heat transfer in experimental conditions. First, the geometry was descripted as follows. The field was a 5-mm-diameter sphere (approximately 65.4 μL in volume) at the centre of a 40-mm-diameter 3-turn magnetic coil (Supplementary Fig. 1: left part). Here, the copper pipe of the magnetic coil was 4 mm and 10 mm of the inner diameter and the outer diameter, separately. Consequently, the 3-turn magnetic coil was 40 mm, 60 mm, 32 mm in the inner diameter, the outer diameter, and the height, separately. The air was 100 mm and 100 mm in the diameter and the height, separately. Owing to the spatial symmetry, only half of the study domain is presented (red dashed line; Fig. 1a: left panel), and calculated based on the meshes (Supplementary Fig. 1: right part).

Second, the material settings and algorithm models were illustrated as follows. The relative permeability and relative permittivity were 1 and 1 for the air, copper coil, and metal gallium; 10 (real part) as well as 0.7 (imaginary part) and 1 for iron oxides. The electric conductivity settings were 6.76 × 10^6^ Sm^−1^ ,100 Sm^−1^, and 0 Sm^−1^ for metal gallium, iron oxides, and the air, respectively [33-34]. Other parameters, such as their density, thermal conductivity, and constant pressure heat capacity, followed the built-in values. The algorithm contained two parts: one was Ampere’s law with 1000 A in coils and the ball; the other was heat transfer in solid with the environment temperature of 293.15 K and heat transfer coefficient of 5 Wm^−2^K^−1^. Considering that the largest *f* × *H* value attained using the AC magnetic field generator with 69.7 nF (Supplementary Fig. 2), the strength *H* and frequency *f* of the AC magnetic field were set at 46652.63 Am^−1^ and 37.2 kHz, respectively, and their product (H × f) of 1.73 × 10^9^ Am^−1^S^−1^ was smaller than the safety criterion of 5× 10^9^ Am^−1^S^−1^. The overall time
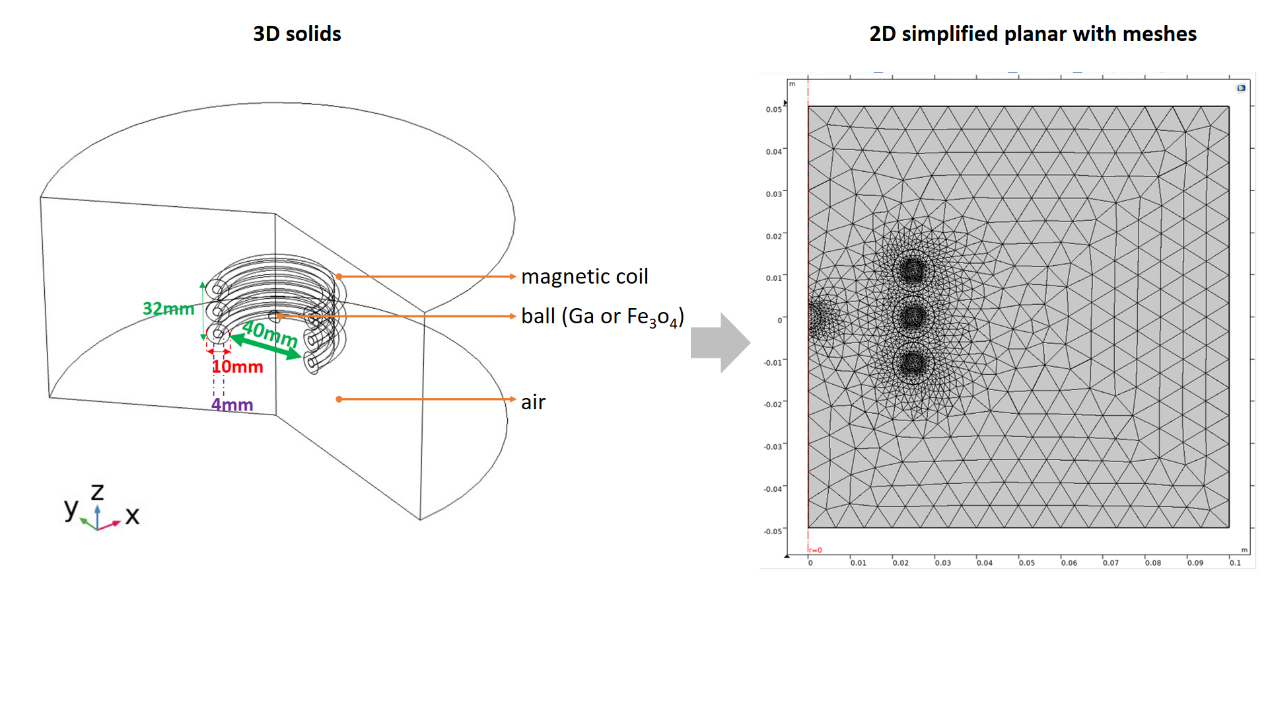
was 10 s according to the time step of 1 s.

**Supplementary Fig. 1** The simulation of the 3D geometry solid and 2D simplified plan with meshes.

- **In-vitro testing**

The utilized platform of the AC magnetic field generator was the modification of the capacity sub-unit based on the high frequency induction heating machine (SP25A, Shenzhen Shuangping Power Supply Technologies Co. Ltd., China) (Supplementary Fig. 2).

With different capacitor modules of 55.7 and 69.7 nF, the strength (H) and frequency (f) of the generated magnetic field was as shown in (Supplementary Fig. 3a). Even though the frequency (f) slightly decreased, the product (f ╳ H) increase with the strength (H) increase. With the module of large electric capacities, the frequency (f) in all AC magnetic fields could be modulated based on the principle of the resonance angular frequency, defined as the inverse proportion to the square root of the product of inductances and capacities (Supplementary Fig. 3a).

The first in-vitro testing for the comparison with the simulation testing was a 65-μL sample of gallium (5N, Kunshan Zhangpu Town Weiju Trading Firm, Kunshan, China) and iron oxides (Fe_3_O_4_, , USA), separately distributed in the same 5-mm-diameter circle on tissue phantoms inserted in a 5-cm-diameter coil centre (Fig. 2b). Here, the tissue phantom was composed of approximately 33.5% w/w boric acid, 35.8% w/w guar gum, 22.9% w/w water, 6.7% w/w polyacrylamide, and 1.1 %w/w triglyceride.

The second type of in-vitro testing was metal gallium in the glass tube (5.9mm, Sinosun Impex Co. LTD, China). The temperature response of different gallium amount showed the large H (i.e. large f ╳ H) increased the rate of temperature increase and highest stable temperature for few amount of metal gallium, such as 59 and 118 μl, but did not for the more one of 177 μl (Supplementary Fig. 3b). The same optimal temperature at 370 ℃ was achieved in different time, such as the application time 23 s for 177 μl at H_1_ (13810.87 Am^-1^ at 49 kHz), and both 14 s for 177 μl and 36 s for 118 μl at H_2_ (14001.26 Am^-1^ at 47.7 kHz). Here, the optimal temperature was limited by the detection range of the optical-fiber temperature sensor. Summarily, the rate of temperature increase increased with both the amount of metal gallium and the applied H (i.e. f ╳ H), rather than f because f decreased at the same. And the amount of metal gallium over the skin depth of several sub-millimeters still contributed the heat generation without the saturation phenomenon.


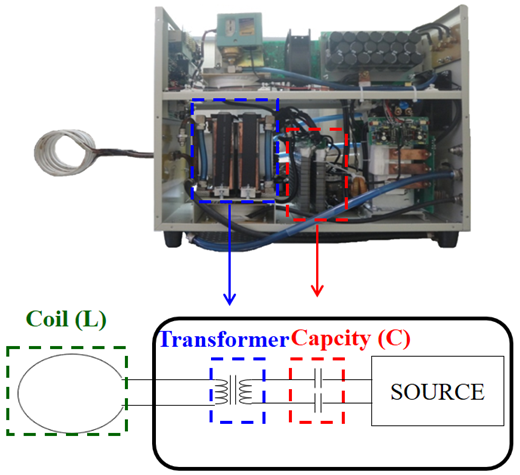


**Supplementary Fig. 2** The modified induction heating machine. The scheme of electronic circuits and the internal photo. The resonance frequency and magnetic field with the modulation of capacity modules.


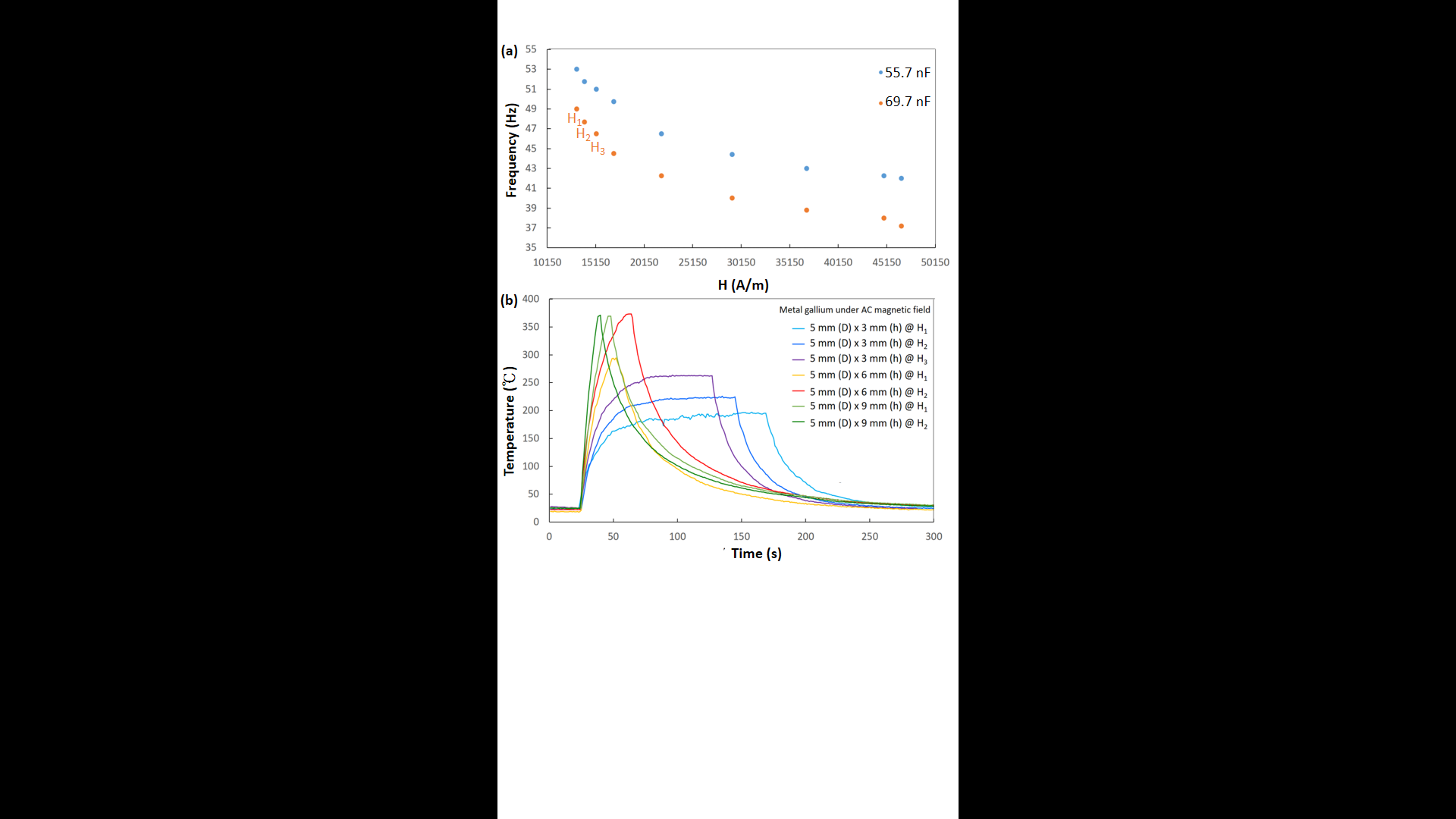


**Supplementary Fig. 3 |** **The temperature response with the applied fields and the amount of liquid metal gallium. a.** The temperature response of the liquid metal gallium of 65 μL under the varied strength (H) and frequency (f) of AC magnetic fields due to homemade capacitor modules of 55.7 nF and 69.7 nF; **b.** The temperature response of liquid metal gallium in different volume (59, 118, 177 μl) under different AC magnetic fields (H_1_: 13810.87 A/m at 49 kHz, H_2_: 14001.26 A/m at 47.7 kHz, H_3_: 15231.84 A/m at 46.5 kHz).

■**Animal testing**

The three parts of animal testing were sequentially executed for three objects: the determination of the suitable ablation time range under the size limitation of rat livers, the validation of various imaging compatibilities, and the validation of stable retention characteristics. And their protocol focuses were listed as follows (Supplementary Fig. 4).


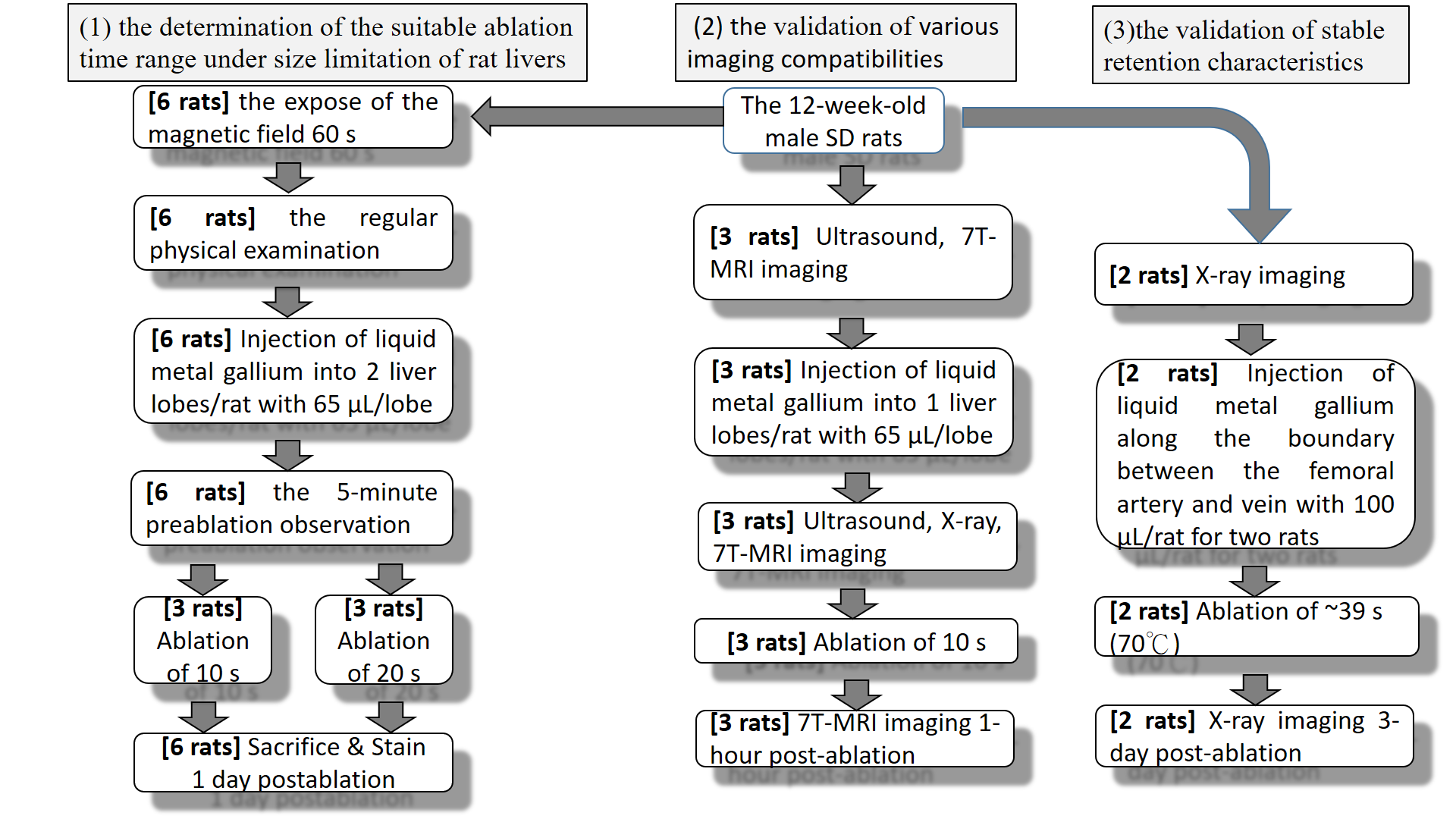


**Supplementary Fig. 4** The protocols for all parts of the animal testing.

■**Degradation testing**

The identification of degraded gallium powders was validated by the ray diffraction (XRD) pattern by powder diffraction file (PDF) number 00-054-0910. The composition was gallium oxide hydroxide (GaOOH), i.e. precursor of Ga_2_O_3_ (Supplementary Fig. 5). The formation was as follows:

Ga^3+^+2H_2_O→GaOOH + 3H^+^


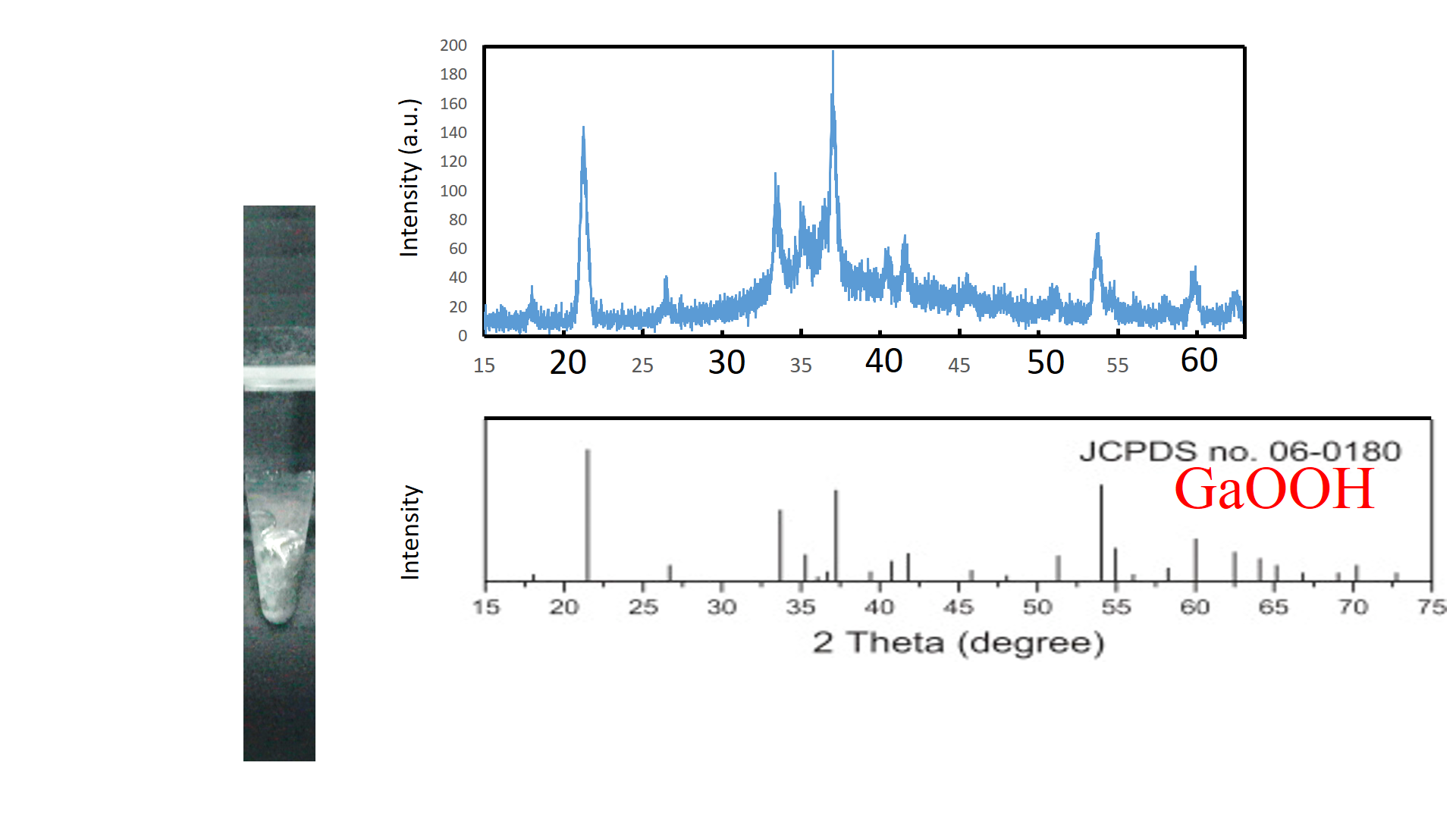


**Supplementary Fig. 5** The identification of composition materials of degraded gallium. The validation of GaOOH by PDF number 04-010-9481.
